# Angina Bullosa Hemorrhagica of the Oral Cavity: A Case Report

**DOI:** 10.7759/cureus.52666

**Published:** 2024-01-21

**Authors:** Eman J Alsheerawi, Mubarak M Alahmed, Sana S Alhewaizem, Motasem M Salih, Reem A Alfayez

**Affiliations:** 1 General Practice, Dream Reem Medical Center, Muharraq, BHR; 2 General Dentistry, Dr. Janat Khalil Dental Center, Isa Town, BHR; 3 General Practice, Al-Hokail Specialist Medical Academic Clinic Company WLL, Hidd, BHR; 4 Dentistry, Dream Reem Medical Center, Muharraq, BHR

**Keywords:** hard and soft palate, hemorrhagic blister, painless blister, oral cavity, angina bullosa hemorrhagica

## Abstract

Angina bullosa hemorrhagica (ABH) is a rare, benign condition that is characterized by a blood-filled blister in the oropharynx. This report describes the case of a 27-year-old male who developed a blister in the oral cavity that ruptured abruptly and resolved spontaneously. The diagnosis of ABH was made according to the diagnostic criteria of Ordioni et al. The core motive of this report is to demonstrate the clinical features and course of ABH to avoid misdiagnosis.

## Introduction

Angina bullosa hemorrhagica (ABH) is a rare condition that leads to recurrent hemorrhagic bullae in the mucosa of the oropharyngeal region due to multifactorial causes [1]. ABH is also associated with several medical conditions, such as hypertension, diabetes mellitus, drug-induced thrombocytopenia, chronic use of steroid inhalers, and masticatory trauma [2]. The bullae enlarge rapidly and then rupture abruptly within 24 to 48 hours. The healing process usually takes 3-10 days, and the condition resolves spontaneously without any further intervention. Although the prevalence of ABH is uncertain, a Brazilian retrospective cross-sectional study of patients with oral and maxillofacial lesions reported a prevalence rate of 0.18% from a total of 12,727 lesions [3].

## Case presentation

A 27-year-old Ugandan male with no medical history presented with the sudden onset of a blister over the hard and soft palates lasting for one hour. Before the formation of the blister, the patient reported eating bread, which scratched the roof of his pharynx, and consuming hot tea. Then he gargled with salt and hot water. Afterward, a painless blister was noticed. The patient reported difficulty in verbal communication, forcing him to communicate through written text, as well as a suffocation-like sensation. This was the patient’s first episode of such swelling. The patient had no chronic bleeding disorders (Table 1) and was not on any regular medications, including blood thinners. The patient had no family history of any chronic diseases.

**Table 1:** Patient laboratory investigations.

Laboratory test	Value	Unit	Reference range
Hemoglobin	15.2	g/dL	13.5–17.5
Platelet count	158	X10^9^/L	150–450
Partial Thromboplastin Time	33.8	Seconds	26–40
Prothrombin Time	12.4	Seconds	11.7–14.2
INR	1.01	-	0.87–1.12
HB A1C%	4.7	%	4–5.7
Fasting glucose	88	mg/dL	70–100

**Figure 1:**
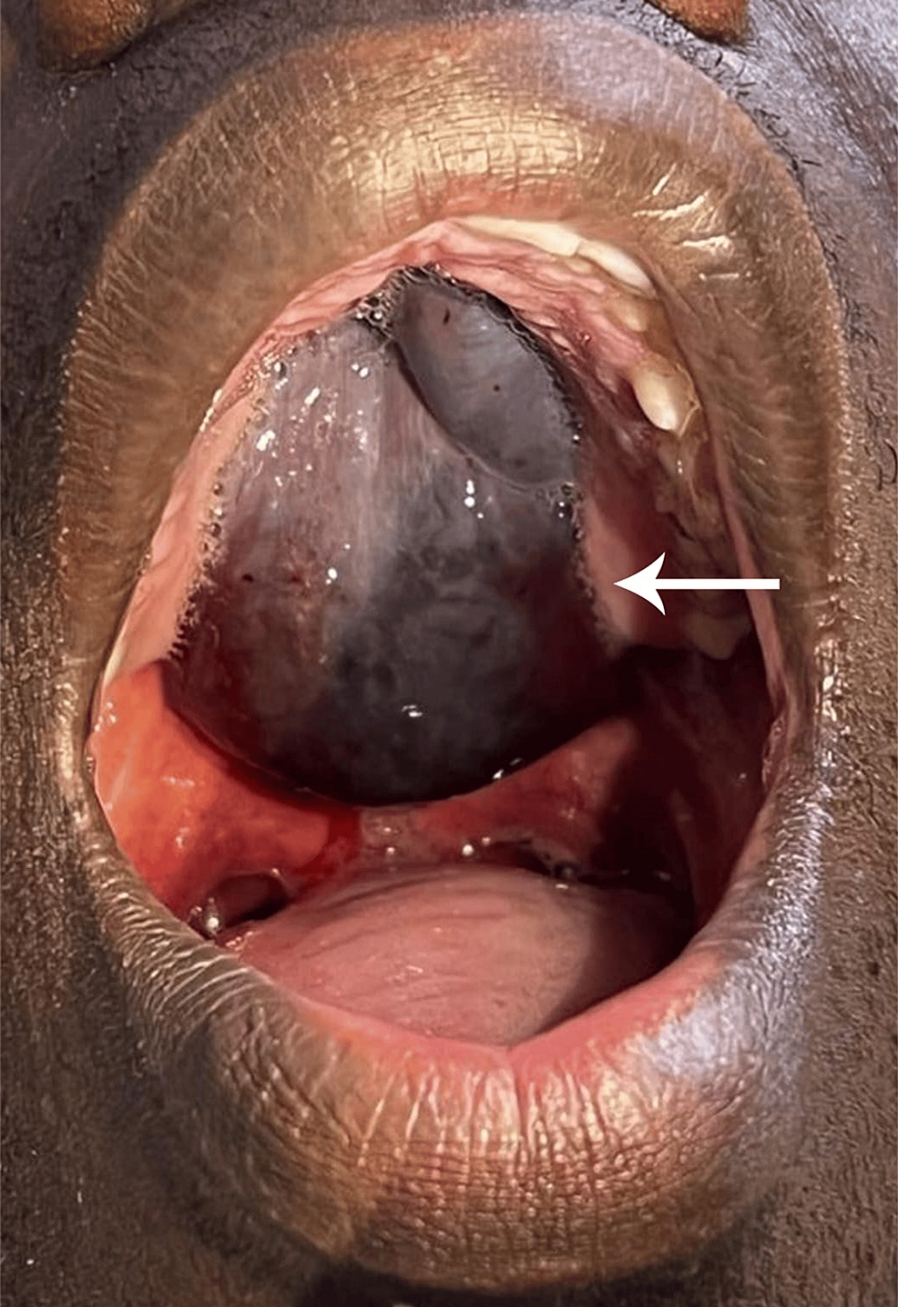
Hemorrhagic blisters on the hard and soft palates before rupture.

Upon examination, the patient’s vital signs were stable. Oral examination revealed a well-circumscribed dark-red blister, 5 cm x 3 cm in size, localized on the hard and soft palates (Figure 1).

**Figure 2:**
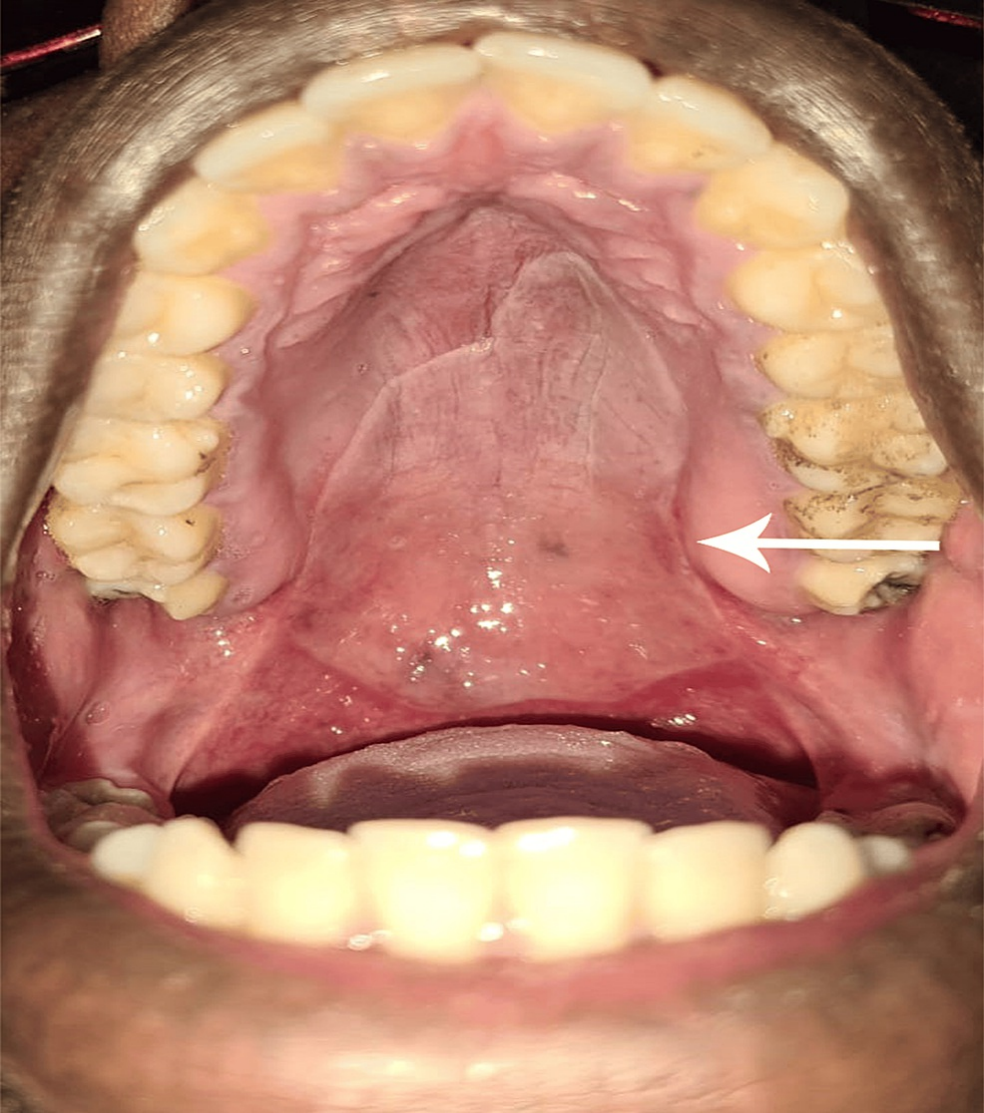
Hemorrhagic blisters occur six hours post-rupture.

On palpation, the blister felt soft, smooth, and non-tender. Laboratory investigations showed a normal platelet count (158 x 10^9^/L) and a normal coagulation profile (Table 1). The blister ruptured abruptly within 2 hours while the patient was in the emergency room. Afterward, the patient was able to speak normally and was discharged. Six hours post-discharge, the patient was reassessed, with examination showing demarcation of the ruptured blister with no active bleeding (Figure 2).

**Figure 3:**
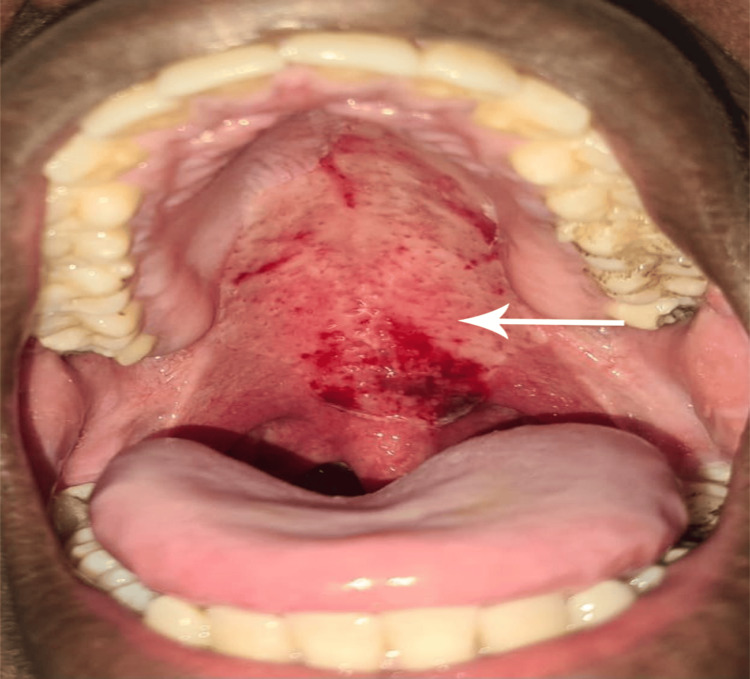
Hemorrhagic blisters three days post-rupture.

The patient was given 0.2% chlorhexidine digluconate mouthwash for five days. The patient had a follow-up visit on day three post-rupture of the blister (Figure 3).

**Figure 4:**
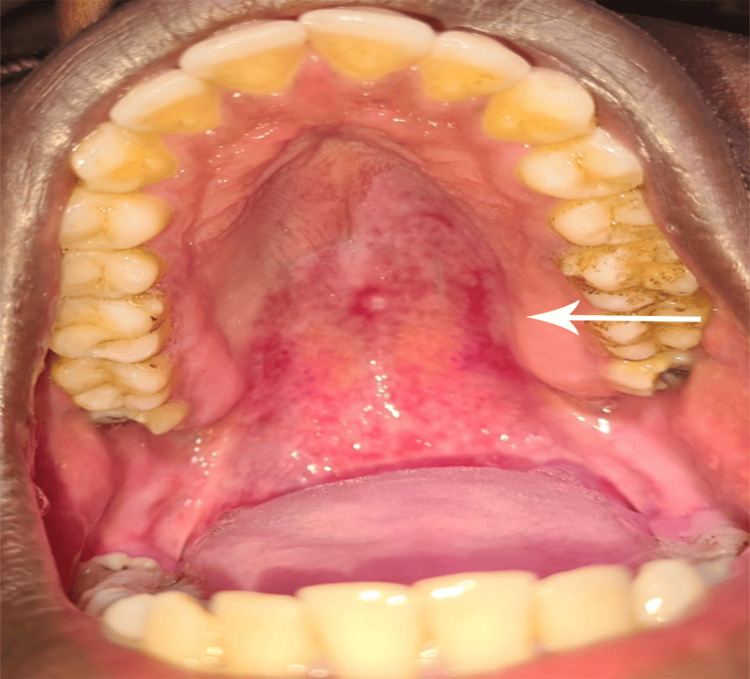
Hemorrhagic blister 10 days post-rupture.

On examination, a shallow, non-tender, erythematous erosion was noted at the same site with multiple blood spots. On day 10, the patient was re-evaluated, and the erosion was expected to fully heal within five months. The patient had no other active complaints (Figure 4).

## Discussion

ABH, which is also known as stomatopompholyx hemorrhagica, benign hemorrhagic bullous stomatitis, and oral hemophlyctenosis, was first described in 1967 by Badham as blood-filled blisters in the oral, oropharyngeal, and/or esophageal mucosa [4]. ABH can occur in the structures of the oropharyngeal cavity, including the soft palate, hard palate, cheeks, floor of the mouth, anterior pillar of the tonsillar fossa, epiglottis, arytenoids, and the esophagus, but it most commonly manifests in the soft palate [5]. The blister tends to spontaneously rupture 1-2 days following its formation [2]. The etiology of ABH is idiopathic and considered multifactorial [6]. Additionally, diabetes mellitus, hyperglycemia, and a family history of diabetes or hypertension are also potential risk factors [1,5,6]. In our case, the patient had a non-tender, large (5 x 3 cm2) ABH covering the hard palate mainly and the soft palate minimally, which resulted in dysphagia, hoarseness, and dyspnea. The patient is known to frequently consume hot tea. The diagnosis of ABH was established according to the criteria recently proposed by Ordioni et al. [7]. The patient had six out of the nine criteria mentioned below (≥6 needed for diagnosis) (Table 2), including two of the main criteria (i.e., clinically noticeable hemorrhagic bulla or erosion with a history of bleeding of the oral mucosa and exclusively oral or oropharyngeal localization), as well as four additional criteria (i.e., palatal localization, triggering event or promoting factor (food intake), painless lesion, tingling, or burning sensation, and normal platelet count and coagulation profile).

**Table 2:** Diagnostic criteria for angina bullosa hemorrhagica (ABH) by Ordioni et al.

Main criteria
(I) Clinically noticeable hemorrhagic bulla or erosion with a history of bleeding of the oral mucosa
(II) Exclusively oral or oropharyngeal localization
Additional criteria
(III) Palatal localization
(IV) Triggering event or promoting factor (food intake)
(V) Recurrent lesions
(VI) Favorable evolution without leaving a scar in a few days
(VII) Painless lesion, tingling, or burning sensation
(VIII) Normal platelet count and coagulation profile
(IX) Negative direct immunofluorescence

A biopsy was not done, and a diagnosis was made clinically. The patient was managed symptomatically until the blister spontaneously ruptured; then, anti-septic rinses were given to prevent secondary infections. Multiple case reports have discussed ABH, one of which is similar to our case in terms of clinical presentation, where a 45-year-old man presented with a sudden-onset blood-filled blister over the soft palate that occurred after eating a meal; the blister ruptured after a few hours and resolved in two days without any scarring [8]. In another case report, a 35-year-old female developed ABH, which became an emergency due to the development of a blister in the back of the throat, leading to dysphagia that was triggered after food ingestion. This case was managed in the ambulance by intravenous hydrocortisone and clemastine [9]. In another case report, a 41-year-old female developed ABH four weeks after being diagnosed with COVID-19; the patient had no triggers or comorbidities and was managed conservatively with a local steroid, chlorhexidine mouthwash, and a multivitamin supplement [10].

## Conclusions

ABH of the oral cavity is considered a rare medical condition. Abrupt blister development in the oral cavity might be frightening at first glance. However, a good approach that involves detailed history-taking in addition to a proper physical examination can lead to the correct diagnosis of ABH. The aim of this paper is to raise awareness about ABH, which will aid in its early recognition and intervention.
